# The clinical presentation of *Legionella* arthritis reveals the mode of infection and the bacterial species: case report and literature review

**DOI:** 10.1186/s12879-019-4488-z

**Published:** 2019-10-21

**Authors:** Marine Ibranosyan, Laetitia Beraud, Hélène Lemaire, Anne-Gaëlle Ranc, Christophe Ginevra, Sophie Jarraud, Ghislaine Descours

**Affiliations:** 1 Hospices Civils de Lyon, Groupement Hospitalier Nord, Institut des Agents Infectieux, Laboratoire de Bactériologie, Lyon, France; 2 Hospices Civils de Lyon, Groupement Hospitalier Nord, Institut des Agents Infectieux, Centre National de Référence des Légionelles, Lyon, France; 3 Hospices Civils de Lyon, Groupement Hospitalier Sud, Hôpital Lyon Sud, Service de Rhumatologie, Pierre-Bénite, France; 4 Hospices Civils de Lyon, Groupement Hospitalier Centre, Hôpital Edouard Herriot, Service de Rhumatologie, Lyon, France; 5 CIRI, Centre International de Recherche en Infectiologie, Equipe Pathogénèse des Légionelles, Lyon, France; 6 Inserm, U1111, Université Lyon 1, CNRS, UMR5308, École Normale Supérieure de Lyon, Lyon, France; 7 Univ Lyon 1, Lyon, France

**Keywords:** *Legionella bozemanii*, Arthritis, Immunosuppression, Corticosteroid, Tocilizumab, Methotrexate, Inoculation, 16S rRNA PCR, Extrapulmonary infections

## Abstract

**Background:**

While *Legionella* is a common cause of pneumonia, extrapulmonary infections like arthritis are scarce. Here, we describe a case of monoarthritis due to *Legionella bozemanii*, with no history of pneumonia. We provide a literature review of the 9 previously published *Legionella* arthritis and highlight a dichotomous epidemiology suggesting different physiopathological pathways leading to joint infection.

**Case presentation:**

A 56-year old woman under immunosuppressive treatment by oral and intra-articular corticosteroids, methotrexate, and tocilizumab for an anti-synthetase syndrome was hospitalized for worsening pain and swelling of the left wrist for 3 days. Clinical examination showed left wrist synovitis and no fever. The arthritis occurred a few days after an accidental fall on wet asphalt responsible for a cutaneous wound followed by a corticosteroid intra-articular injection. Due to both the negativity of conventional culture of articular fluid and suspicion of infection, 16S rRNA and specific PCRs were performed leading to the identification of *L. bozemanii*. *Legionella*-specific culture of the articular fluid was performed retrospectively and isolated *L. bozemanii*. The empiric antibiotic therapy was switched for oral levofloxacin and rifampin and the patient recovered after a 12-week treatment.

**Conclusion:**

We report a case of *L. bozemanii* monoarthritis in an immunosuppressed woman, following a fall on wet asphalt and intra-articular corticosteroid injection. The review of the literature found that the clinical presentation reveals the mode of infection and the bacterial species. Monoarthritis more likely occurred after inoculation in patients under immunosuppressive therapy and were associated with non-*Legionella pneumophila* serogroup 1 (Lp1) strains that predominate in the environment. Polyarthritis were more likely secondary legionellosis localizations after blood spread of Lp1, the most frequently found in pneumonia. In both settings, 16S rRNA and *Legionella*-specific PCR were key factors for the diagnosis.

## Background

Legionnaires’ disease (LD), a severe pneumonia caused by *Legionella*, represents 2–9% of cases of community-acquired pneumonia. There are more than 70 species and serogroups, but *Legionella pneumophila* serogroup 1 (Lp1) is responsible for more than 85% of cases of LD worldwide, while non-Lp1 infections occur more frequently in immunosuppressed patients [1]. Extrapulmonary infections, including arthritis, are scarce and not systematically related to pneumonia, suggesting a multi-faceted infectious process. Herein, we report a case of *L. bozemanii* monoarthritis in an immunosuppressed woman, following a fall on wet asphalt and intra-articular corticosteroid (CS) injections.

## Case presentation

A 56-year-old woman was hospitalized in the rheumatology department for worsening pain and swelling of the left wrist over the 3 preceding days. Her medical history included type 1 diabete and anti-synthetase syndrome diagnosed 1 year previously and which was characterized by polyarthritis, dermatomyositis and interstitial lung disease, and treated by prednisone 10 mg/day, methotrexate (MTX) 20 mg/week, and tocilizumab 560 mg/month. During the 2 previous months, she suffered from stage-II synovitis of the left wrist predominant at radiocarpal and middle carpal joints, associated with flexor and extensor digitorum communis tenosynovitis; she received 2 intra-articular injections of triamcinolone 2 months and 3 days before admission.

**Fig. 1:**
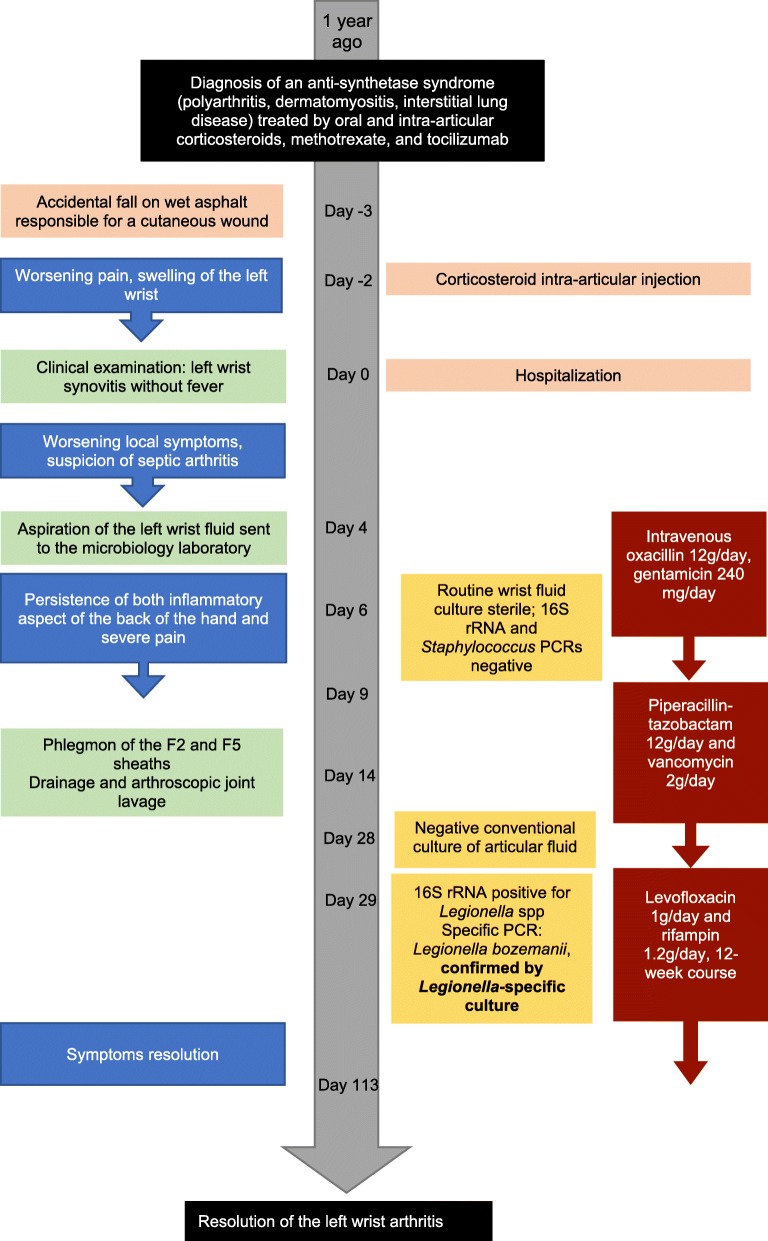
Timeline of interventions and outcomes

On admission, the patient showed left wrist synovitis with no fever. Clinical examination found a wound of the 3rd proximal interphalangeal joint, related to an accidental fall on wet asphalt 4 days before. The blood count and C-reactive protein were normal. Chest X-ray showed no worsening of the interstitial lung disease. Blood samples were collected for culture. Over the following days, while she remained afebrile, local symptoms worsened, with onset of a painful epitrochlear lymphadenopathy. On day 4, a septic arthritis was suspected and aspiration of the left wrist fluid was performed; hemorrhagic fluid with a white blood cell count of 15.7 G/L and a neutrophil count of 8.0 G/L (51%) was found. One sample was sent to the microbiology laboratory; Gram stain revealed no microorganism. An intravenous anti-staphylococcal antibiotic therapy (oxacillin 12 g/day, gentamicin 240 mg/day) was initiated. On day 6, routine wrist fluid culture remained sterile, 16S rRNA [2] and *Staphylococcus* PCRs were negative, and blood cultures also remained sterile. On day 9, while the patient had improved, an inflammatory aspect of the back of the hand and severe pain persisted. The antibiotic spectrum was broadened to piperacillin-tazobactam 12 g/day and vancomycin 2 g/day on infectiologist advice. On day 14, the patient had not improved and she underwent emergency surgery that found phlegmon of the F2 and F5 sheaths, requiring drainage and arthroscopic joint lavage. Six samples were sent to the microbiology laboratory; after 14 days of culture (day 28) a single *Lysinibacillus fusiformis* colony was found in 1 sample, and rare *Cutibacterium acnes* colonies in a second sample, which were considered as contaminants. On day 29, the 16S rRNA PCRs were positive for *Legionella* spp. for 2 of the 4 sterile samples. The 23S–5S PCR performed on these samples identified *L. bozemanii*. The antibiotic regimen was modified to a 12-week course of oral levofloxacin 1 g/day and rifampin 1.2 g/day, and the symptoms quickly resolved. The immunosuppressive therapy by prednisone and MTX was continued Fig. 1.

Retrospectively, the joint fluid sampled on day 4 also tested positive for *L. bozemanii* by 23S–5S PCR. Five specimens (1 collected on day 4, and 4 on day 14) were plated onto BCYE, BMPA and MWY plates (Oxoid, Dardilly, France). Only 1 day-14 sample grew *L. bozemanii* after 9 days. A *L. bozemanii* seroconversion (< 1/16 before admission, 1/256 on day 53) was demonstrated by in-house serology.

Regarding the source of infection, no clinical or radiological pneumonia was diagnosed before or during the course of the infection. *L. bozemanii* inoculation from asphalt at the time of her fall was strongly suspected. We hypothesized that the immunosuppressive treatment contributed to development of the infection and delayed diagnosis.

## Discussion and conclusions

When examining this case alongside those previously published and summarized in Table 1 (*n* = 9) [3–11], it is of note that *Legionella* arthritis was mostly identified incidentally by 16S rRNA PCR, which is consistent with the characteristics of the bacterium that does not usually grow on standard media. Interestingly, 2 clinical presentations can be distinguished. The first is monoarthritis (*n* = 7) that were all due to non-Lp1 strains and mainly reported in immunosuppressed patients (*n* = 6) with no history of pneumonia (*n* = 7); in 5 of the 7 cases, skin trauma close to the affected joint (*n* = 2) or an intra-articular injection or joint surgery (*n* = 3) was reported before arthritis. In contrast, cases of polyarthritis (*n* = 3) were all due to Lp1 and observed in patients with no immunosuppressive treatment who presented pneumonia at the time or before the diagnosis of arthritis. This dichotomous epidemiology suggests different physiopathological pathways leading to joint infection, which may result from both strain- and patient-related factors.

**Table 1:** Demographic characteristics, clinical features, and microbiological diagnoses of the case reports of *Legionella* arthritis, including the current case

Case	Gender	Age (y)	Localization	Strain	Microbiological diagnosis	Medical history	Immunosuppressive treatment	Suspected origin of the infection	Pneumonia
Current case	Female	56	Left wrist	*L. bozemanii*	16S rRNA PCR, culture	Anti-synthetase syndrome	Oral and intra-articular CS, MTX, tocilizumab	Wound after fall on a wet asphalt	No
Banderet et al. 2017 [3]	Female	90	Left wrist	*L. cincinnatiensis*	16S rRNA PCR, culture	Presumed chondrocalcinosis, chronic kidney disease	Oral and intra- articular CS	Intra-articular CS injection	No
Just et al. 2012 [4]	Female	71	Left knee	*L. bozemanii*	16S rRNA PCR, culture	Dermatomyositis	Oral CS, MTX	Skin biopsy	No
Fernández-Cruz et al. 2011 [5]	Female	83	Right knee	*L. micdadei*	16S rRNA PCR, culture	Rheumatoid arthritis	Oral CS, MTX	Replacement of prosthesis the year before	No
Flendrie et al. 2011 [6]	Female	58	Right knee	*L. dumoffii*	16S rRNA PCR, culture	Systemic erythematosus lupus-like disease	Oral and intra-articular CS, MTX	Intra-articular injection 2 days before	No
Dugar et al. 2009 [7]	Male	56	Left foot	*L. longbeachae*	Culture	Rheumatoid arthritis, diabetes mellitus	Oral CS, MTX	Unknown	No
Linscott et al. 2004 [8]	Female	80	Right MCP joint	*L. pneumophila* serogroup 4	Culture on chocolate agar	No	No	Unknown	No
Thurneysen and Boggian 2014 [9]	Male	70	Right knee Left ankle	*L. pneumophila* serogroup 1	16S rRNA PCR, culture	Secondary thymoma- associated immunoglobulin deficiency	No	Unknown	Yes, 5 months before
Naito et al. 2007 [10]	Female	80	Left and Right ankle	*L. pneumophila* serogroup 1	Urinary antigen, 16S rRNA PCR	Chronic kidney disease	No	Unknown	Yes, LD 16 days before
Bemer et al. 2002 [11]	Male	51	Right wrist and ankle, both knees	*L. pneumophila* serogroup 1	Urinary antigen, Culture on mycobacteria medium	Recurrence of thymoma 1 year before	No	Unknown	Yes, at the same time

*CS* Corticosteroid, *LD* Legionnaires’ disease, *MCP* Metacarpophalangeal, *MTX* Methotrexate, *rRNA PCR* ribosomal ribonucleic acid polymerase chain reaction

While more than 70 *Legionella* species have been isolated from freshwater and soil environments, less than half have been observed in clinical settings, and Lp1 is responsible for the vast majority of LD cases worldwide [1]. In contrast with pneumonia, non-Lp1 *Legionella* strains are predominant among the cases of monoarthritis reported in the literature (Table 1). This particular epidemiology is consistent with the environmental distribution of *Legionella* strains [12] and the direct mode of transmission from their natural niche; a similar epidemiology has been described for skin and soft tissue *Legionella* infections [13].

Host response to *Legionella* infections involves both innate and adaptive immunity [14, 15] and immunosuppressive therapies such as systemic CS, cytotoxic chemotherapies, and biological therapies (i.e. tumor necrosis factor inhibitors) are risk factors for *Legionella* infections [15–17]. Post-inoculation arthritis were described in patients receiving CS with or without MTX (Table 1), suggesting that inoculated *Legionella* was not cleared at the first step of infection. Three cases received intra-articular CS that constitutes both a gateway for environmental germs and an additional local risk factor for infection. In the case described herein, the patient also received tocilizumab, an IL-6 receptor antagonist. A case of Lp1 pneumonia in a patient under tocilizimab has been described [18]. As described herein, the patient presented no fever as tocilizumab inhibits IL-6, an inflammation and fever mediator [19], and, by delaying the diagnosis, tocilizumab contributes to uncontrolled infections.

Another clinical feature of *Legionella* arthritis is polyarthritis occurring during the course or after pneumonia. Interestingly, the reported cases are all due to Lp1 strains that predominate in LD (Table 1) suggesting secondary joint infections after bacterial blood dissemination. Accordingly, cases of Lp1 bacteremia have been described [20–22], and Lindsay et al. reported that up to 80.5% of patients present an Lp1 blood positive PCR after LD onset [23]. Nevertheless, non-Lp1 bacteremias have also been described [24, 25] and no data comparing blood bacterial loads between Lp1 and non-Lp1 strains are available.

In conclusion, the clinical presentation of *Legionella* arthritis reveals the mode of infection and orientates the microbiological diagnosis towards either Lp1 or non-Lp1. In both contexts, 16S rRNA and *Legionella* specific PCRs are key factors for the diagnosis.

## Data Availability

Not applicable.

## Abbreviations

CS: Corticosteroid
LD: Legionnaires’ disease
Lp1: *Legionella pneumophila* serogroup 1
MCP: Metacarpophalangeal
MTX: Methotrexate
rRNA PCR: Ribosomal ribonucleic acid polymerase chain reaction
